# Optimization of Bone Cement Stiffness in Metastatic Vertebral Augmentation: Balancing Strength Restoration and Stress Redistribution

**DOI:** 10.1007/s10439-025-03948-z

**Published:** 2025-12-17

**Authors:** Mehran Fereydoonpour, Asghar Rezaei, Lichun Lu, Mariusz Ziejewski, Ghodrat Karami

**Affiliations:** 1 Department of Mechanical Engineering, North Dakota State University, Fargo, ND 58108-6050 USA; 2 Department of Physiology and Biomedical Engineering, Mayo Clinic, Rochester, MN USA

**Keywords:** Vertebral augmentation, Bone cement, QCT/FEA, Stress redistribution, Patient-specific modeling, Defect size, Optimization

## Abstract

**Purpose:**

The goal of this study was to investigate the mechanical performance of vertebral augmentation with various polymer-based materials across different defect sizes. Specifically, this study aimed to identify the optimal stiffness of bone cement that maximizes vertebral strength while minimizing stress redistribution.

**Method:**

A calibrated quantitative computed tomography-based finite element analysis (QCT/FEA) approach was developed and calibrated against cadaveric experimental data. Lytic metastatic defects were simulated in human vertebrae at two augmentation volumes (20 and 50%) and filled with materials spanning a wide range of elastic moduli (50 to 2500 MPa). Stress distributions and fracture forces were analyzed in six vertebrae to evaluate the influence of material stiffness and augmentation size.

**Results:**

The QCT/FEA models accurately predicted vertebral strength (R^2^ = 0.96) and showed that increased material stiffness leads to higher fracture force but also significantly elevates stress concentrations. An augmentation material with an elastic modulus of approximately 300 MPa offered a favorable balance between strength restoration and minimal stress elevation, especially for 50% augmentation size. Paired t-tests revealed that materials with moduli ≤ 300 MPa did not produce statistically significant stress redistribution compared to intact bones, while stiffer materials (≥1000 MPa) did.

**Conclusions:**

The findings suggest that a bone cement stiffness of approximately 300 MPa may provide optimal mechanical benefits by enhancing vertebral strength without inducing excessive stress redistribution. The study also highlights that augmentation size strongly influences the mechanical outcomes, with larger augmentation volumes showing greater sensitivity to material stiffness. The proposed patient-specific QCT/FEA framework provides a cost-efficient, adaptable tool for preclinical evaluation and personalized planning of vertebral augmentation These insights can assist material developers in optimizing bone cement formulations for patient-specific treatments.

## Introduction

Vertebral augmentation has become a widely accepted treatment for managing osteoporotic and metastatic vertebral fractures [1]. This procedure, which involves injecting bone cement into weakened vertebrae, aims to restore mechanical stability, alleviate pain, and reduce the risk of further collapse. [2]. One of the key biomechanical outcomes of vertebral augmentation is the alteration of stress distribution within the treated vertebra and its neighboring vertebrae [3]. While effective in improving immediate structural support, the procedure can also introduce stress concentration changes that affect adjacent vertebrae, potentially leading to complications [4, 5].

Historically, polymethylmethacrylate (PMMA) has been the most commonly used augmentation material due to its ease of handling and high stiffness [6, 7]. However, this high stiffness can lead to stress shielding of the augmented vertebra, thereby altering the load path and creating higher focal points of loading in adjacent levels. As a result, several studies have associated PMMA augmentation with adjacent-level vertebral fractures. For instance, excessive stiffness from PMMA cements has been associated with heightened stress in adjacent vertebrae, increasing the risk of subsequent fractures as well as disc extrusion and other forms of disc degeneration [3, 8].

To mitigate these complications, researchers have explored the use of alternative materials with a wider range of mechanical properties. Materials such as calcium phosphate cements [9], bioactive glass [10], and composite formulations have been developed in order to better match the mechanical behavior of cancellous bone and reduce complications associated with conventional cements [11]. Some studies suggest that the choice of material (e.g., PMMA vs. Cortoss) does not significantly alter the overall mechanical restoration achieved through vertebroplasty [12, 13]. Although multiple studies have investigated fracture outcomes after augmentation, most studies have not focused on stress redistributions within the vertebral body because of augmentation [14]. While different augmentation materials have been proposed and investigated by multiple studies [15], it remains unclear which material properties are most suitable for providing sufficient structural support while minimizing internal stress distributions. These findings underscore the need for deeper investigation using computational tools such as FEA, which can simulate and quantify how different materials and augmentation sizes influence fracture and stress patterns within the vertebral body and neighboring segments. Moreover, for such analyses to be effectively translated into clinical settings, methods must be developed that are patient-specific, require minimal manual effort, and can be readily integrated into routine medical imaging workflows.

The current study employs QCT/FEA to assess how augmentation performed in idealized locations with materials of varying elastic modulus affects the mechanical properties and fracture behavior of the vertebra. Therefore, the aims of the current study are twofold. The first aim is to identify the optimal augmentation material that can restore the structural integrity of the bone by comparing five different materials. The second aim is to assess mechanical stress redistributions within vertebral bodies superior and inferior to the augmented region. Through this, the study identifies a material with an optimal elastic modulus that provides strength while minimizing internal stress to reduce the risk of stress shielding.

## Materials and Methods

An integrated experimental–computational approach has been employed to investigate the biomechanical performance of vertebral augmentation using polymers of varying stiffness. One key parameter examined in this study is the influence of vertebral defect size. This section outlines the experimental setup and computational methodology.

The primary objective was to assess how different augmentation materials and lesion sizes affect vertebral strength and internal stress distribution. The computational model was constructed using QCT imaging data to simulate vertebral defects and their augmentation with polymers spanning a range of elastic moduli. Model calibration was performed using experimental data from cadaveric vertebral specimens subjected to in vitro mechanical testing.

Following successful calibration, the QCT/FEA model was employed to simulate intact vertebral bodies (*n *= 6) with artificially induced defects of two sizes (20 and 50%), augmented using materials with stiffness values ranging from 50 to 2500 MPa. The experimental procedures, modeling approach, and calibration techniques are detailed in the following sections.

In this study, the term fracture force is used to denote the maximum load a vertebra can withstand before structural collapse. Although in classical engineering mechanics the term fracture often implies crack propagation, here the term refers to the peak load at which a sudden and substantial drop in the force–displacement response is observed.

### Experimental Setup

The imaging and mechanical testing data were obtained from a previous study including details of experimental [16]. The experimental component of this study was approved by the Mayo Clinic Institutional Review Board through its Biospecimens Subcommittee. The cadaveric spinal columns were obtained from nine donors (5 male) with an average age of 73 ± 12 years. The demographic information is given in Table 1.

**Table 1:** Donor demographic information and vertebral levels used in the study. The listed intact vertebral specimens were used in both the experimental calibration tests and the corresponding QCT/FEA simulations

No.	Bone ID	Group	Level	Sex	Age	*BMD* $gr/c{m}^{2}$
1	5082T10	Defect	T10	M	68	0.993
2	5110T6	Defect	T6	F	91	0.455
3	5166T6	Defect	T6	M	60	0.519
4	5166T12	Defect	T12	M	60	0.605
5	5118T9	Intact	T9	F	69	0.843
6	5133T6	Intact	T6	M	67	0.675
7	5186T8	Intact	T8	M	81	0.622
8	5107T6	Intact	T6	F	74	0.445
9	5133T9	Intact	T9	M	67	0.71
10	5166T9	Intact	T9	M	60	0.611
11	5107T12	Augmented	T12	F	74	0.408
12	5110T9	Augmented	T9	F	91	0.502
13	5154T7	Augmented	T7	F	95	0.533
14	5166L3	Augmented	L3	M	60	0.697
15	5186T5	Augmented	T5	M	81	0.653

Three groups of specimens were included in this study: intact, simulated defect, and augmented. All experiments and analyses were conducted on the vertebral body with the posterior elements removed. To experimentally create the defect and augmented specimens, anterior lesions were simulated by drilling holes of carefully controlled diameter and depth into the vertebral bodies [16]. A subset of these specimens was subsequently augmented using poly(propylene fumarate) (PPF), which is a novel biodegradable biomaterial. To facilitate imaging and mechanical testing, the superior and inferior ends of each vertebral body were embedded in PMMA. QCT imaging was performed using a Siemens CT scanner (Siemens Healthcare GmbH, Germany) with settings of 120 kVp, 200 mAs, and a resolution of 0.586 mm × 0.586 mm × 0.6 mm, including a calibration phantom. These QCT datasets were used to reconstruct the 3D geometry of each vertebra and assess its mineral density distribution for further modeling.

Another component of the experimental setup involved mechanical testing using an MTS 858 Mini Bionix II system (MTS Systems Corporation, Eden Prairie, MN). During testing, the inferior PMMA block was rigidly fixed to the base of the testing machine, while the superior PMMA block remained free to move. A spherical loading crosshead applied a vertically directed displacement at a controlled rate of 5.0 mm/min. The spherical interface allowed the specimen to move vertically as prescribed, while permitting unconstrained motion and rotation in other directions, thereby minimizing artificial constraints. Force–displacement data were recorded continuously throughout the test. The fracture force was defined as the point at which a notable drop in load was observed, indicating structural collapse of the specimen.

### Computational Procedure

#### QCT/FEA Methodology

**Fig. 1:**
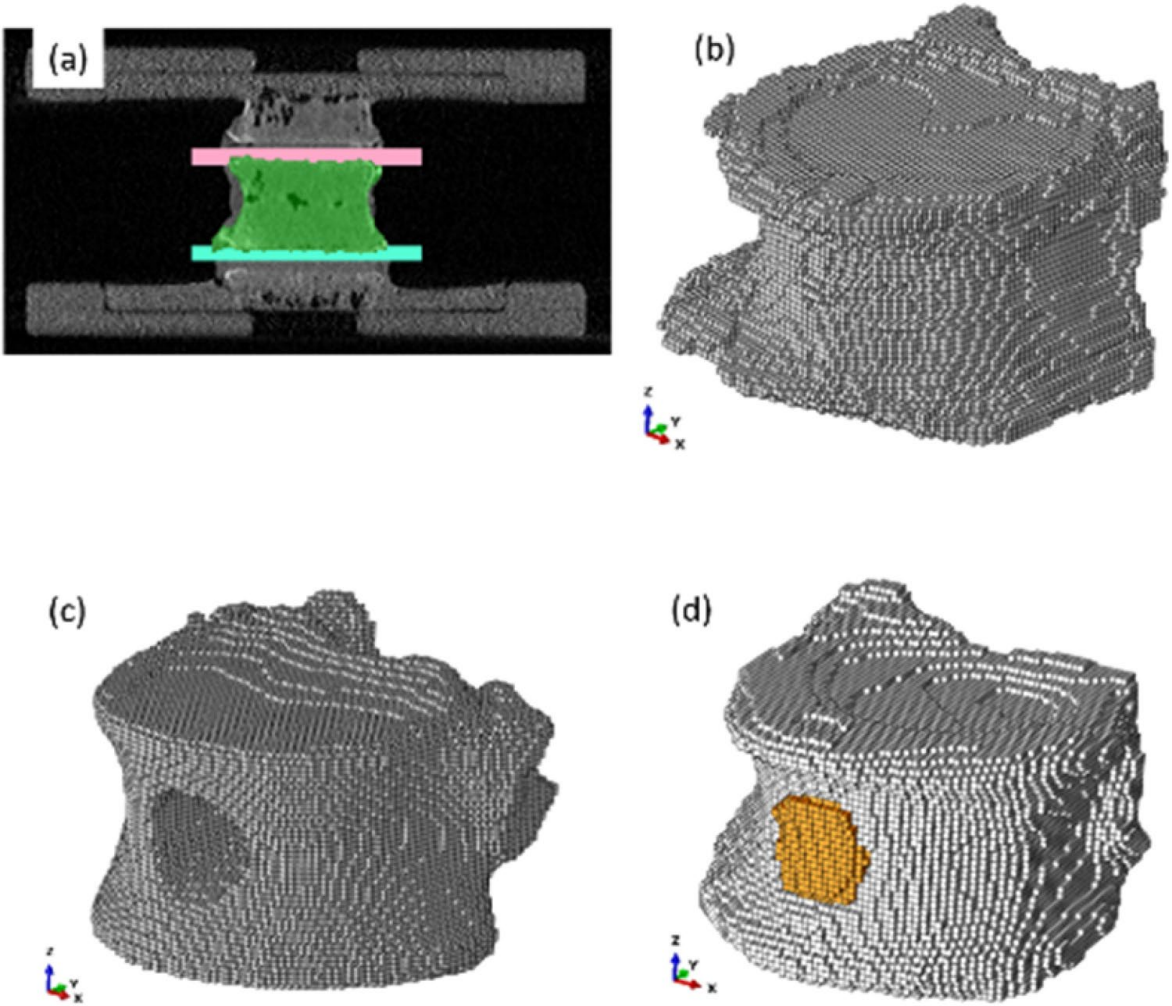
**a** QCT image segmentation of the vertebral body performed in Mimics. Manual segmentation was applied to isolate the central vertebral body and reconstruct its full geometry. Two thin rectangular volumes were then created above and below the vertebra to capture the superior and inferior PMMA block interfaces. The vertebral geometry was subtracted from these blocks to generate accurate footprint surfaces at the top and bottom of the vertebra. Representative voxel models are shown for **b** intact vertebra, **c** vertebra with a simulated defect, and (d) vertebra augmented with biomaterial (orange)

The process for constructing the QCT/FEA models has been described in detail in a previous study [17]; a summary is provided here for convenience. QCT imaging data were used to generate 3D geometry of the vertebral bone through segmentation using Mimics software (Materialise, Ann Arbor, MI), Figure1a. This process produced a voxel-based model with voxel dimensions corresponding to the smallest resolution of the CT scanner, as noted in the previous section. In addition to geometric information, each voxel was assigned a bone mineral density (BMD) value derived from its Hounsfield Unit (HU) by referencing calibrated phantom values. To assign material properties to the finite element models, QCT-derived BMD values were converted to apparent density (ρ) and subsequently to elastic modulus (E) using phenomenological power-law relationships of the general form $E=a\cdot {\rho }^{b},$ where *a* and *b* are empirical coefficients. We calibrated the coefficients a and b for each specimen (Table S1). Calibration was performed by minimizing the difference between the experimental fracture force and the FE-predicted fracture force for that specific specimen. This ensured that each model accurately reproduced the mechanical response of the corresponding vertebra before simulating augmentation conditions. [16]. No additional correction factors were applied for metastatic tissue, as the calibration was performed on vertebrae with induced defects rather than clinical lesions. Subsequently, an elastic modulus was assigned to each voxel using the established density–modulus conversion equation, allowing spatial heterogeneity in material properties. A uniform Poisson’s ratio of 0.3 was applied to all voxels in the model, consistent with prior literature [17].

The FEA models were developed in ABAQUS software (Version 2024, Dassault Systèmes Simulia Corp., Johnston, RI, USA). The models utilized C3D8 elements, which are 8-node, linear, three-dimensional hexahedral solid elements with 2 × 2 × 2 integration points. These elements represented both the bone and the superior and inferior PMMA blocks. The element size was matched exactly to the voxel dimensions derived from the QCT data, allowing for a direct translation of image-based geometry into the mesh. This voxel-based meshing approach preserved the original shape and size of each voxel, enabling a geometrically faithful model that better captures local variations in material properties and promotes higher accuracy in mechanical predictions.

#### Fracture Model

Fracture modeling was implemented using a strain-based progressive failure criterion, using the empirical relationship:${\varepsilon }_{yield}=0.0081 {\rho }^{-1.42} ,$ where ${\varepsilon }_{yield}$ denotes the yield strain threshold for that voxel and ρ was the ash density [16]. During the simulation, for each increment of applied displacement, the strain in every element was computed and compared to the corresponding yield strain of each element. If the local strain exceeded the yield threshold, the element was considered to have failed. To simulate post-yield behavior, the elastic modulus of the failed element was reduced to a nominal value of 0.1 MPa, effectively removing its load-bearing capacity [17].

#### Loading and Boundary Conditions

To mimic the loading and boundary conditions of the experimental setup, the computational models were run under pure compression loading. The boundary conditions for all nodes on the lower plane of the inferior PMMA considered fixed in all directions, while the superior PMMA was subjected to prescribed displacement in vertical direction, with no constraints applied to its movement in other degrees of freedom, resembling the boundary conditions on testing machine.

To calibrate the finite element modeling, the QCT/FEA-predicted fracture force values were compared with their corresponding experimentally measured values in each group. Figure 1 shows one model from each experimental group. The vertebral body with simulated defect contains a hole created by drilling which is shown in the finite element model (Figure 1c). The augmented bone was modeled as shown in Figure 1d. Here, the hole is filled with elements those have the properties of the augmented material (PPF in this study). By setting the boundary conditions for the finite element model same as experimental testing and solving the model, the results of the finite element model were calibrated against the experimental output.

#### Simulated Defect and Augmentation Modeling

**Fig. 2:**
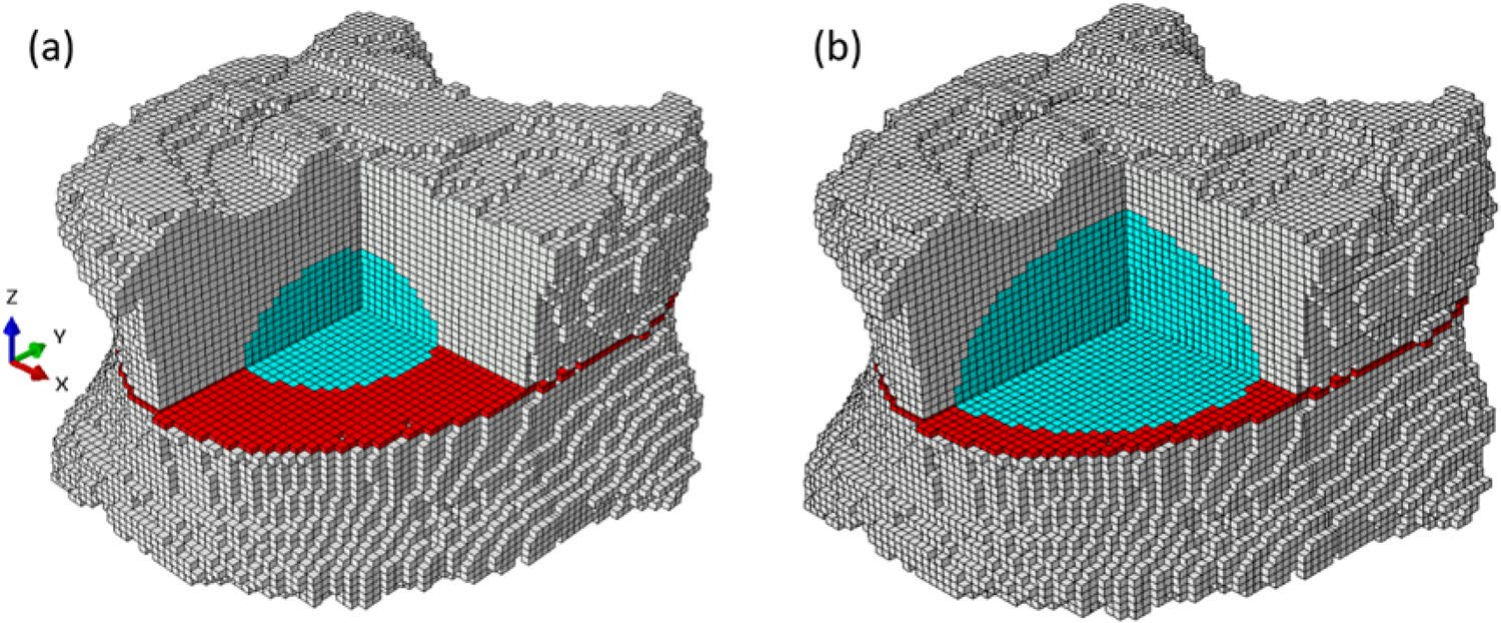
Voxel-based model of a vertebral body with simulated defect volumes. The vertebra is shown in gray, and the red plane represents the anatomical plane parallel to the transverse plane at the level of minimum bone cross-sectional area. The cyan regions represent the simulated defect (augmentation) volumes inside the bone. **a** Model with a 20% defect and **b** model with a 50% defect. This visualization highlights the geometric positioning of the defects relative to the bone structure and reference plane

After calibration, the intact vertebral bodies were chosen to investigate augmentation performance of different materials. The defect (and augmentation) shape was assumed elliptical at the center of the intact vertebral body [17]. The tumor size was defined as the percentage of the tumor’s cross-sectional area in the transverse plane (Figure 2) [18].

In both the calibration study and the augmentation models, lesion regions were assigned a nominal elastic modulus of 0.1 MPa, effectively eliminating their load-bearing capacity and simulating failed elements within the vertebra. Two defect sizes were investigated: 20% and 50%. Additionally, five different elastic moduli were assessed to define augmentation materials. These values were selected to represent a wide spectrum of augmentation materials: 50 MPa, corresponding to Vertecem® PMMA modified with 50 vol.% hydroxyapatite (which reduces its elastic modulus from 1,840 to 50 MPa [19]); 300 MPa, representing low-modulus PMMA bone cement [20]; 1,000 MPa, corresponding to dicalcium phosphate dihydrate (DCPD) composite cement [21]; 2,000 MPa, corresponding to poly(propylene fumarate) (PPF); and 2,500 MPa, corresponding to conventional PMMA bone cement. These values cover a broad range of augmentation material properties representative of clinical and experimental biomaterials [11]. For each vertebra, one intact model and two defect models (20% and 50%) were generated, resulting in 18 models across the cohort. Each defect case was further augmented with the five different materials, leading to 10 augmented models per vertebra and 60 augmented models in total. Altogether, this yielded 78 finite element models (6 intact, 12 defect, and 60 augmented) used for the analysis.

**Fig. 3:**
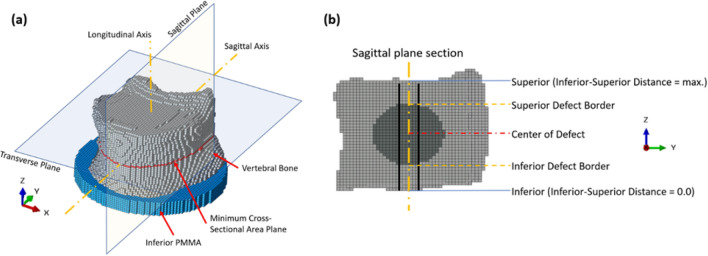
**a** Vertebral bone 3D view, with inferior PMMA, **b** Section of the bone in Sagittal plane, with elliptical augmentation, the line in orange is Longitudinal axis

To investigate stress distributions, a midsagittal anatomical plane was selected to obtain von Mises stress values (Figure 3a). This plane allows visualization of stress variation along vertical (longitudinal) axis, which aligns with the primary direction of compressive loading in the spine. The direction along which stress values were extracted is shown in Figure 3b. In the midsagittal plane, stress data were collected along the three lines (two black lines, and one center line) shown in Fig 3b. The distance of these adjacent lines from the longitudinal axis was set to three times the element length in the sagittal direction. The stress values of elements at the same vertical level were then averaged to evaluate the stress distribution profile. The compressive force used to generate this stress distribution for each bone corresponds to the maximum load that the bone with the largest defect (50%) can tolerate before fracture.

#### Statistical Analysis

Statistical analyses were conducted using the Statisty online statistical calculator. Mean values and standard deviations for fracture forces were calculated for both the experimental and computational datasets. Mean values for maximum stresses were calculated for the computational dataset. Linear regression analysis was performed to evaluate the correlation between experimentally measured fracture forces and their corresponding QCT/FEA-predicted values. To determine the impact of augmentation materials on internal stress distribution, paired t-tests were conducted comparing the stress results for each augmentation material against those of the intact condition for each vertebra. The normality of the maximum stress data was assessed using the Shapiro–Wilk test. In addition, the paired differences used for the t-tests were also checked for normality using the Shapiro–Wilk test.

Because multiple vertebrae originated from the same donors, we also performed linear mixed-effects analyses to account for donor clustering. A mixed model was fit with MaxStress as the outcome, Condition, Group (20%/50%) and Region (Upper/Lower) as fixed effects, and Donor as a random intercept. Intraclass correlation coefficient (ICC) was computed from the estimated variance components.

## Results

### Calibration of Computational Modeling

To assess the accuracy of the FEA model, the predicted fracture forces were compared with the experimental fracture forces measured for each vertebra included in the study.

These data demonstrate the variability in vertebral strength across different bones and form the basis for the calibration of the computational modeling described in the following section.

For each sample, specimen-specific density–elasticity coefficients were calibrated. Across all vertebrae, the coefficient *a* had a mean of 7546, standard deviation of 3179, and range from 928 to 11230, while the exponent *b* had a mean of 2.00, standard deviation of 0.42, and range from 1.39 to 2.84.

**Fig. 4:**
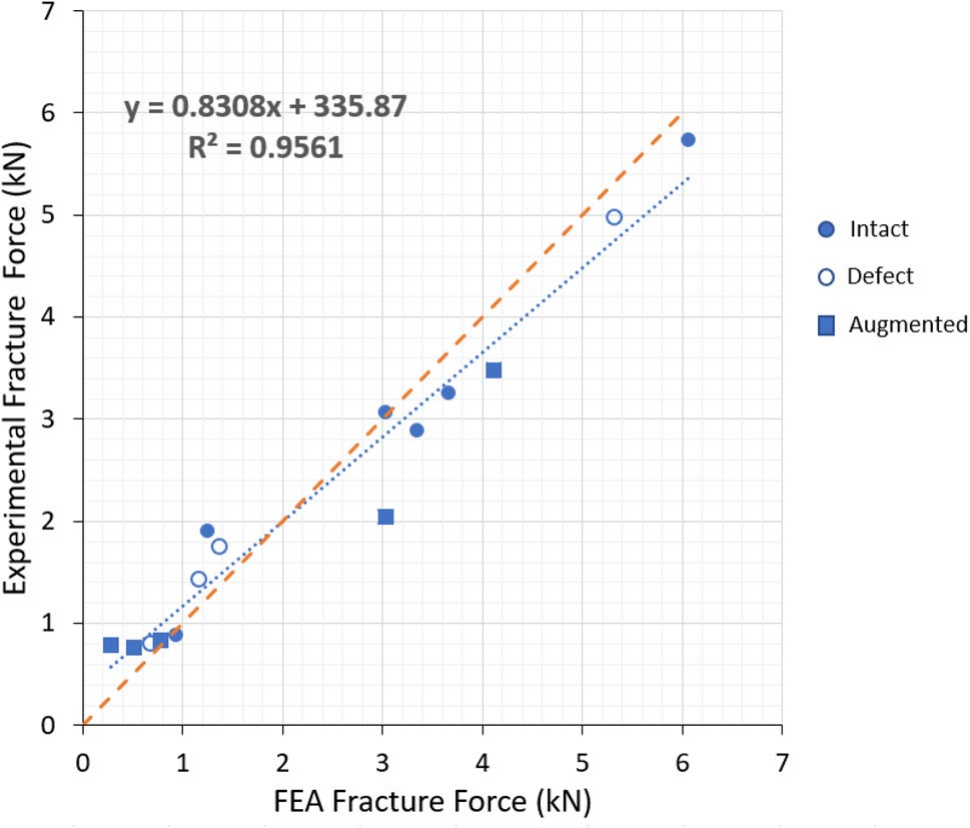
Calibration of the QCT/FEA models against experimental fracture force. The horizontal axis shows the FE-predicted fracture force obtained using specimen-specific calibrated density–elasticity coefficients ***a*, *b***, while the vertical axis shows the experimentally measured fracture force from cadaveric testing. The dashed line represents the ideal 1:1 correspondence

Linear regression analysis was performed between the experimental fracture loads and their computational counterparts, revealing a strong agreement, with a correlation coefficient of 0.95 (p < 0.001), indicating excellent predictive accuracy of the computational model (Figure 4).

### Vertebral Strength Response to Augmentation

**Fig. 5:**
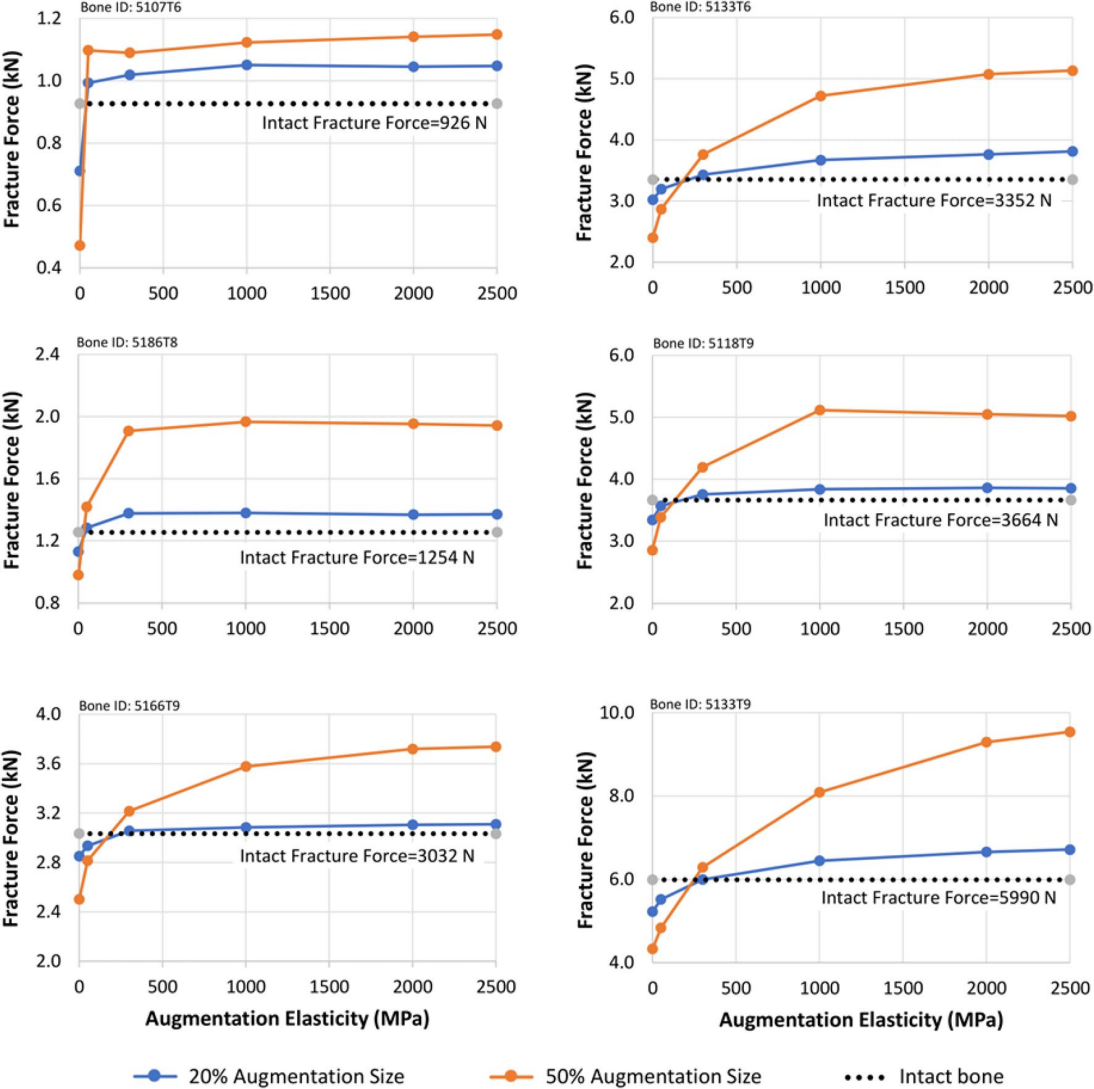
Computationally predicted fracture forces for different augmentation materials across multiple vertebral specimens. Each plot corresponds to a specific vertebra, evaluated under three conditions: intact (0% tumor), indicated by a black horizontal dashed line; 20% tumor, shown in blue; and 50% tumor, shown in orange. The x-axis represents the elastic modulus of the augmentation materials, while the y-axis shows the predicted fracture force

The computational results are presented in Figure 5. Each graph corresponds to a specific vertebral body, arranged in ascending order of their intact vertebral strength. In all cases, augmentation with materials of an elastic modulus of 300 MPa or greater resulted in a predicted fracture force exceeding that of the intact specimen.

In all specimens, the augmentation material with an elastic modulus of 300 MPa restored structural integrity to a level equal to or greater than that of the intact condition, regardless of the augmentation size. The weaker specimens (e.g., 5107T6 and 5186T8) did not benefit from the use of stiffer augmentation materials, whereas the stronger specimens (e.g., 5118T9) showed increased strength with higher elastic moduli.

### Vertebral Stress Response to Augmentation

**Fig. 6:**
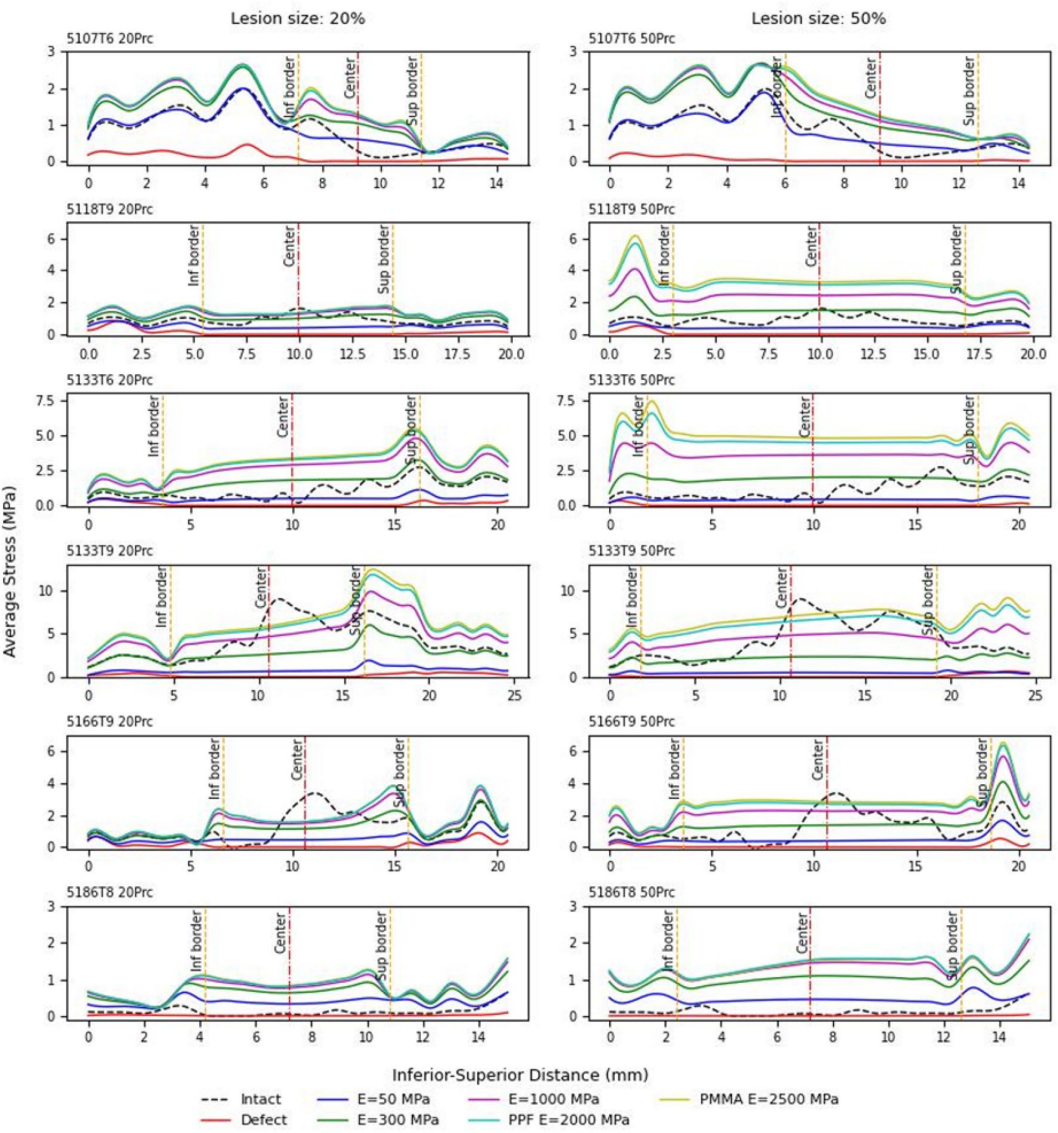
Stress distribution along the vertical axis of all the vertebral specimens for both defect sizes of 20% (shown in the left column) and 50% (shown in the right column). Stresses were extracted along a midsagittal plane using the maximum load tolerated by each bone in the 50% defect condition

Von Mises stress distributions along the longitudinal axis were evaluated for the vertebral specimens (Figure 6). The same amount of load was applied to each vertebral body for both defect sizes, and Von Mises stress values were averaged. Each graph presents seven curves including an intact, a simulated defect and five augmentation materials.

**Fig. 7:**
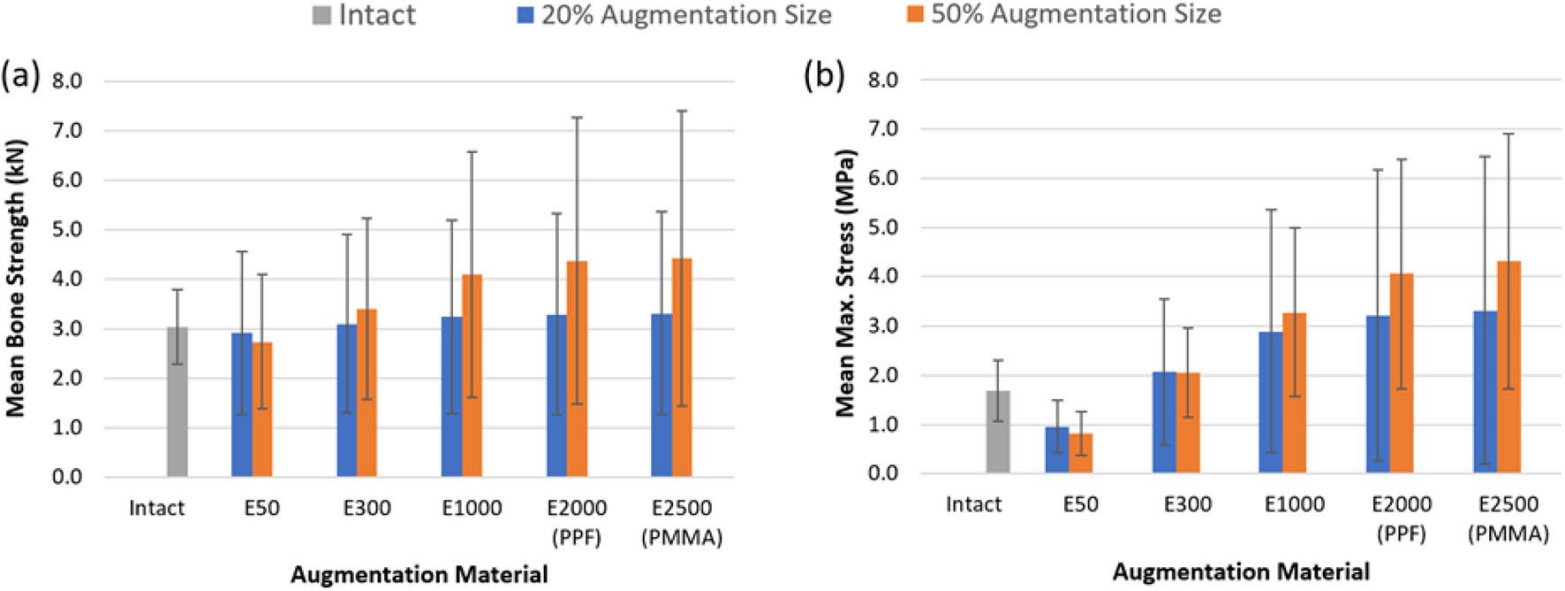
**a**The variation of the mean bone fracture force and **b** the variation of the mean maximum stress for all bones with 20% and 50% augmentation size and different augmentation materials, Intact, E50 (*E* = 50 MPa), E300 (*E* = 300MPa), E1000 (*E* = 1000 MPa), PPF (*E* = 2000 MPa), and PMMA (*E* = 2500 MPa), the standard deviations are shown on each bar

To compare strength and stress concentration effects from different augmentation materials, the average strength (derived from Figure 5) and average maximum stress (derived from Figure 6) across all specimens and augmentation materials are compared in Figure 7.

Increasing the stiffness of the augmentation material from E300 (elastic modulus = 300 MPa) to PMMA (elastic modulus = 2500 MPa) results in an increase in average fracture force from 3409 to 4420 N, corresponding to a 29.6% gain in strength (Figure 7a). Concurrently, the mean maximum stress rises from 2.18 to 4.94 MPa, amounting to a 106% increase (Figure 7b).

The paired t-test results indicated that there were no statistically significant differences between the intact condition and either E50 (*p* = 0.114) or E300 (*p* = 0.225). This means that for these materials, the null hypothesis of no difference could not be rejected. In contrast, statistically significant differences were found when comparing the intact condition with E1000 (*p* = 0.006), PPF (*p* = 0.011), and PMMA (*p *= 0.015), indicating that these augmentation materials produced stress distributions that differed significantly from the intact state. These results suggest that lower-stiffness augmentation materials (E50 and E300) behave more similarly to intact bone in terms of stress distribution, whereas higher-stiffness materials (E1000, PPF, PMMA) lead to altered distributions.

When donor clustering was accounted for using a linear mixed-effects model, donor variance was 0.738 (residual variance 2.238) yielding an ICC ≈ 0.25, indicating that ~ 25% of the variance is attributable to donor. After adjusting for donor, the model showed that E1000 (*p* = 0.0014), PPF (*p* < 1e−5), and PMMA (*p* < 1e−5) produced significantly higher stresses than Intact, while E50 (*p* = 0.068) and E300 (*p *= 0.380) were not significantly different from Intact. These mixed-model results support the main findings from the paired tests and confirm that the conclusions are robust to donor clustering.

**Fig. 8:**
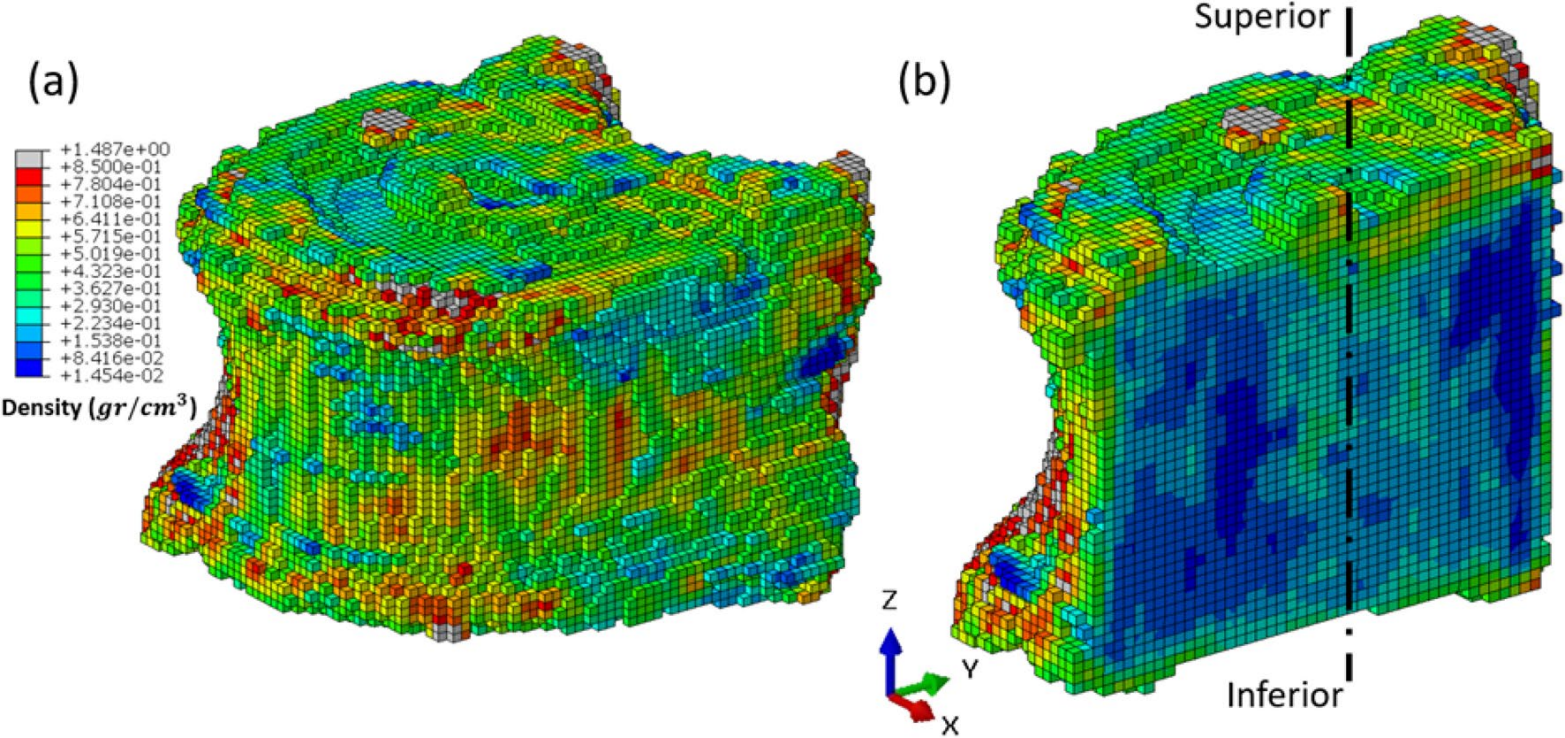
**a** Three-dimensional voxel-based representation of vertebra 5133T9 showing the contour distribution of bone density across the structure. **b** Sagittal plane section of the same vertebra illustrating the spatial variation in density along the superior–inferior axis, highlighting regions of locally elevated density embedded within lower-density areas. This heterogeneity reflects the intrinsic bone microstructure and contributes to specimen-specific stress distribution patterns

A sagittal section of the intact vertebra 5133T9 was examined to visualize its density distribution over the voxel mesh. The resulting map is shown in Figure 8 and illustrates the spatial variability in density across the bone along the inferior–superior axis. This visualization helps explain specimen-specific variations in stress distribution observed between the intact and augmented cases.

## Discussion

This study employed a computational model to assess vertebral fractures in both intact and augmented vertebral bodies. The findings highlight the critical role of the bone's baseline structural integrity (the strength of vertebra in intact condition) in determining the effectiveness of augmentation [22]. Although increasing augmentation stiffness increased vertebral fracture strength, the rate of improvement plateaued beyond approximately 1000 MPa, meaning that further increases in stiffness produced only modest additional strength gains while substantially elevating internal stress levels. While initial increases in augmentation stiffness substantially improved fracture force, further increases offered progressively smaller gains, especially in the weaker specimens, which are clinically more relevant. Our findings suggest that PMMA is excessively strong for augmentation purposes, generating stress concentrations in the bone adjacent to the augmentation material. A material with an elastic modulus of 300 MPa provides sufficient structural integrity to the vertebral body while minimizing stress concentrations. In contrast, materials with an elastic modulus greater than 1000 MPa do not necessarily offer additional strength but instead induce large stress concentrations, which may damage the trabecular structures superior and inferior to the augmented region. Therefore, an elastic modulus in the range of 300 to 1000 MPa can be considered a safe and effective choice for augmentation, offering optimized strength restoration while minimizing stress concentrations.

We used specimen-specific calibration because no consensus density–elasticity law exists for vertebral bone, and published power-law coefficients vary widely across studies. As shown in the comprehensive review by Helgason et al. [23], experimentally derived relationships of the form $E=a{\rho }^{b}$ report a varying by more than 60-fold and b ranging from ~ 1.2 to 3.0. Because no universally accepted relationship exists for vertebral bone, recent studies have recommended individualized relationships to avoid propagating systematic modeling bias (e.g., Eberle et al. [24]). In our study, we required the density–elasticity law to be accurate for each individual specimen, because our overarching goal was to evaluate the biomechanical effect of a range of polymer stiffnesses, not to test a specific density–elasticity law. Therefore, specimen-specific calibration minimized the confounding effect of incorrect modulus assignment and improved the reliability of the subsequent augmentation-related comparisons. Only the elastic modulus coefficients (*a*, *b*) were calibrated. The yield–strain relationship was identical for all specimens, ensuring that failure behavior was driven by density patterns rather than individualized strain limits.

The calibrated coefficients obtained in this study for the density–elasticity power-law relationship also falls well within the broad ranges reported in the literature. As summarized in the review by Helgason et al. [23], vertebral trabecular bone exhibits substantial variability in experimentally derived elasticity–density relationships, largely due to differences in testing methodology, specimen geometry, density measurement approaches, and anatomical location. The commonly cited ranges for human vertebral cancellous bone span approximately a = 320–16 600 MPa for the coefficient and *b* = 1.00–2.87 for the exponent in relationships of the form $E = \,a\rho^{b}$. In comparison, the calibrated coefficients obtained in the present work (*a* = 928–11 230; *b* = 1.39–2.84) lie comfortably within these established ranges, indicating that the variability observed here is expected and consistent with previous experimental and QCT-based studies. This agreement further supports the appropriateness of the specimen-specific calibration approach used in this study.

The high R^2^ (0.96) obtained in the calibration reflects the use of specimen-specific coefficients (*a*, *b*) for the density–elasticity relationship. Thus, this value indicates the quality of the calibration rather than universal predictive accuracy. Independent validation on an unseen dataset, without specimen-specific tuning, will be required to assess the model’s generalizability.

Although elastic modulus was calibrated for each specimen, the post-yield behavior was modeled using a single density–yield strain power-law applied uniformly to all vertebrae. This simplification does not capture specimen-specific variability in post-yield response, which may amplify discrepancies between predicted and experimental fracture forces for some specimens. For example, the outlier behavior observed for bone ID 5107T12 may be partially attributable to the use of a non-individualized yield strain formulation. Nonetheless, the primary sources of these deviations are biological variability and limitations inherent to QCT resolution and trabecular microstructure representation, which have been similarly reported in previous QCT/FEA validation studies [14, 25].

Stress is closely related to strain and displacement through constitutive models, and several studies have validated finite element predictions of these measurable quantities using high-resolution techniques such as digital image correlation (DIC) and digital volume correlation (DVC). For instance, Garavelli et al. reported excellent agreement (R^2^ > 0.9, RMSE% < 8%) between DIC-measured displacements and subject-specific FE model predictions of metastatic vertebrae [26]. Palanca et al. demonstrated that micro-FE models reproduced local displacements measured with DVC in vertebrae with induced defects [27]. More recently, Garavelli et al. confirmed good correspondence between homogenized FE models and full-field DVC displacement and strain data in metastatic vertebrae, including the identification of strain concentration regions [28]. Collectively, these studies provide indirect but strong evidence that QCT/FEA models yield realistic biomechanical fields, and therefore, the stress redistributions observed in our simulations likely reflect true mechanical trends.

In this study, we selected a range of elastic moduli spanning from 50 to 2500 MPa to represent the mechanical spectrum of materials used in vertebral augmentation. This selection was informed by the mechanical properties of cancellous bone, which typically exhibits an elastic modulus in the range of 50–500 MPa depending on density and anatomical location [29–31]. On the lower end of our elastic modulus range (50–300 MPa), we included values representative of low-modulus polymer cements, such as brushite or calcium phosphate-based materials, and experimental low-stiffness PMMA formulations, which have been developed to reduce stiffness mismatch with native bone and mitigate adjacent-level fractures [9, 32, 33]. Materials like PPF and modified PMMA cements fall within the 100–2000 MPa range and have been investigated for their improved biocompatibility and tunable stiffness [11, 14]. The upper bound of our range (2500 MPa) represents conventional PMMA cement, which is widely used in clinical practice for its high strength and handling properties, despite concerns over stress shielding and increased risk of adjacent fractures [6, 34]. By encompassing this wide spectrum, our study aims to capture both the clinical relevance and biomechanical implications of using materials with varying stiffness for vertebral augmentation.

Several prior studies have addressed the biomechanical consequences of using high-stiffness bone cements such as PMMA. Boger et al. performed mechanical testing on vertebrae augmented with either high-modulus PMMA cements (elastic modulus 2000–2500 MPa) or lower-modulus cements (~490 MPa). They found that high-modulus cement led to increased stress concentrations in adjacent vertebrae, potentially elevating the risk of adjacent-level fractures [31]. In contrast, the lower-modulus PMMA provided adequate mechanical reinforcement and appeared to reduce stress transfer to adjacent vertebrae, although the study did not directly quantify stress concentrations [31]. Wilcox et al. used a FEA approach to investigate how vertebroplasty using PMMA cement (elastic modulus 2000 ~ 2500 MPa) affects stress distribution in adjacent vertebrae [30]. Robo et al. investigated a novel low-modulus PMMA bone cement and found that it had approximately 77% lower elastic modulus than standard PMMA, while still providing adequate compressive strength for vertebral support and demonstrating good fatigue performance over time [32]. Their results showed that the reduced stiffness minimized the mismatch between the cement and native bone, potentially lowering stress concentrations and the risk of adjacent-level fractures. The findings of our current study are consistent with those of Boger et al., but our computational approach allowed for direct evaluation of stress responses associated with different augmentation materials. We identified ~ 300 MPa as a critical stiffness threshold beyond which further increases in modulus result in diminishing strength gains and increased internal stress. Our analysis encompassed a wider range of materials, including elastic moduli of 50, 300, 1000, 2000, and 2500 MPa. Notably, the 490 MPa cement used in Boger et al.’s study falls between our E300 and E1000 groups, providing experimental support that aligns closely with our proposed optimal range (~300 MPa).

Peng et al. employed a generic FEA model of only a single vertebra and identified an optimal range of bone cement elastic modulus between 833 and 1408 MPa to achieve a balance between vertebral strength restoration and minimizing stress transfer to adjacent vertebrae [35]. This range somewhat aligns with the findings of our study, which suggest a more compliant polymer can be favorable for both strength and stress distributions. Our study, however, employed a calibrated patient-specific computational model with six specimens having a wide range of fracture forces and showed a more compliant polymer with a modulus between 300 MPa and 1000 MPa offers a more biomechanically favorable outcome. The results of our study also indicate that the lower end of this range is more suitable for weaker vertebral bodies or smaller defect sizes, whereas the upper end is more appropriate for stronger bone or larger defects. Clinically, this differentiation between weaker and stronger bone can be guided by BMD values obtained from dual-energy X-ray absorptiometry (DXA) or HU values derived from QCT. These results suggest optimizing cement stiffness on a patient-specific basis, targeting enough stiffness to restore strength without excessive stress shielding

A key finding of this study is the disproportionate relationship between augmentation stiffness and the resulting mechanical outcomes. Specifically, increasing the stiffness of the augmentation material from E300 (300 MPa) to PMMA (2500 MPa) led to a 29.6% increase in average fracture force, while the average stress more than doubled, rising 106%. This indicates that although higher-stiffness augmentation materials such as PMMA provide some strength benefits, they also introduce significantly greater internal stress concentrations, which may outweigh those benefits. These results directly support the core objective of this study, which is to identify augmentation strategies that restore sufficient vertebral strength without exacerbating stress shielding.

The resulting map (Figure 8) revealed marked heterogeneity along the inferior–superior axis, with localized regions of higher density embedded within areas of lower density. This spatial variability in bone density influences the local stress response under loading, leading to specimen-specific differences in stress peaks. In particular, the presence of dense voxel clusters in certain regions of the intact bone can result in locally elevated stresses compared to the more homogenized stress distribution observed after augmentation. This finding highlights the importance of considering intrinsic bone heterogeneity when interpreting stress outcomes and underscores why augmentation may not uniformly reduce peak stresses in all cases. While these factors introduce localized variations, the broader stress patterns and trends captured by the model remain valid for interpreting augmentation effects.

This study has several limitations that should be acknowledged. First, the analysis was based on a limited number of specimens, which may restrict the generalizability of the findings across a broader population with varying bone qualities and anatomical variations. Second, the investigation focused exclusively on axial compressive loading, while other physiologically relevant loading modes, such as flexion, extension, and lateral bending, also contribute to vertebral fracture mechanisms and should be considered in future analyses. Third, the tumor defect was modeled as a single elliptical void, whereas in clinical settings, tumor shapes are often irregular and variable. Additionally, only a single tumor per vertebra was simulated, whereas multiple lesions may coexist and interact biomechanically.

As another limitation, the FE models were calibrated using specimen-specific density–elasticity coefficients to match experimentally measured fracture forces. While this improves accuracy for the present comparative analysis, it also means that the calibrated equations are not directly generalizable to clinical imaging data. Independent validation using an entirely separate dataset, without specimen-specific calibration, will be required to confirm broader applicability of the modeling framework.

A further limitation of this study is that calibration of the computational models was performed on vertebrae with induced artificial defects rather than realistic metastatic lesions. True metastatic lesions are highly heterogeneous and may present with mixed lytic and sclerotic regions, irregular geometries, and altered properties in the surrounding bone tissue. These complexities are not fully captured by clinical CT resolution or by the simplified defect models used here, and therefore, the exact local stress redistributions in metastatic bone may differ from our predictions. Nevertheless, the simplified approach provided a controlled modeling environment, where lesion size, shape, and location could be prescribed systematically. This allowed us to isolate and study the influence of individual parameters in a reproducible way, an approach that is not feasible with naturally occurring lesions where geometry and material changes are uncontrolled. Despite these limitations, the general mechanical trends identified, namely that increasing augmentation stiffness increases vertebral strength but also elevates stress shielding, are expected to remain valid. Future studies incorporating patient-specific reconstructions of metastatic lesions and their associated material heterogeneity will be necessary to extend the applicability of these findings to clinical metastatic cases.

An additional limitation of this study is that the computational models were based on QCT-derived density–elasticity relationships and therefore did not explicitly represent the intrinsic microstructure of vertebral trabecular bone or the complex heterogeneity of metastatic lesions. Clinical CT resolution is not sufficient to capture fine-scale architectural features such as trabecular orientation, sclerotic/lytic subregions, or localized microdamage, all of which may influence local stress distributions and fracture initiation. While prior validation studies have shown that QCT/FEA provides reliable predictions of vertebral strength at the whole-organ level (R^2^ typically > 0.8) despite the lack of microstructural detail, deviations in individual cases, as observed in our cohort, likely reflect this biological variability and modeling simplification. Future work with higher-resolution imaging or advanced constitutive models could help bridge this gap, but at the clinically applicable scale, QCT/FEA remains a practical and robust approach for evaluating augmentation strategies.

A further limitation relates to the experimental setup. Although our calibration was performed on three-level spine segments (Figure 1a) with natural boundary conditions, we did not perform post-fracture CT imaging, which prevents precise determination of the fracture type or localization. In physiologic disc loading, fractures often localize near the lesion–endplate interface, whereas our setup may have influenced the exact fracture patterns. Nevertheless, the overall experimental outcomes were consistent with simulation predictions, both indicating compressive fracture and loss of vertebral strength. Importantly, previous work has shown that single-vertebra QCT/FEA models can still predict fracture outcomes in multi-level spine segments with good accuracy (R^2^ = 0.70–0.84, Rezaei et al. [16]). This supports the robustness of our approach while also highlighting the practical advantages of single-level modeling, which is more efficient, less complex, and more cost-effective for potential clinical applications. Future work should integrate post-fracture imaging and disc–vertebra interactions to refine localization of fracture and strengthen clinical translation.

Although the finite element models were validated against experimental fracture forces, direct validation of internal stress distributions was not possible, since stress cannot be measured experimentally in bone tissue. This represents an inherent limitation in the calibration of our modeling framework. While previous studies have demonstrated strong agreement between FE-predicted and experimentally measured displacements and strains using DIC/DVC techniques, which indirectly support the reliability of stress predictions, these results cannot fully replace direct stress field validation. Future work incorporating DIC/DVC-based experimental methods for displacement and strain mapping could provide an additional layer of verification and increase confidence in the local stress redistribution patterns predicted by QCT/FEA models.

A further limitation is that lesion-induced changes in the surrounding trabecular bone structure were not explicitly modeled. Only the transition between defect and bone material was represented, which may lead to local stress concentrations captured by the FE analysis. Nevertheless, potential adaptive or pathological remodeling around lesions could further influence the mechanical response, and future models should consider these effects.

Another limitation is that the defect regions were modeled as being completely filled with augmentation material, whereas in clinical scenarios residual dense tissue may prevent full cement infiltration or lead to mixing of materials. While this simplification may overestimate the uniformity of augmentation and influence local stress redistribution, it was necessary to enable controlled comparisons of cement volume, stiffness, and defect size, with the recognition that partial filling in clinical reality may alter augmentation outcomes.

Although the modeling in this study was based on high-resolution CT scans (0.6 mm slice thickness) to optimize geometric fidelity and material mapping, clinical CT protocols often use lower resolutions. Previous research has shown that while lower image resolution may reduce the precision of geometry reconstruction and material property assignment, QCT/FEA models can still maintain good predictive value when appropriately calibrated [36]. Therefore, the framework presented here remains clinically relevant, though future work should systematically evaluate its performance across a range of clinically realistic resolutions to fully establish its robustness for clinical translation.

Finally, the loading in this study was assumed to be quasi-static, and dynamic or impact loading scenarios were not addressed. These conditions, which are relevant during falls or sudden movements, may require incorporation of rate-dependent material properties, such as those highlighted by McLean et al., to more accurately capture time-sensitive bone behavior [37].

## Conclusion

This study employed a calibrated QCT/FEA framework to investigate the biomechanical performance of vertebral augmentation using materials with varying elastic moduli and augmentation sizes. The objective was to determine the stiffness thresholds that would restore vertebral strength while minimizing stress redistribution, which is a key factor in adjacent-level fracture risk. The study combined experimental calibration with computational modeling to ensure accurate prediction of fracture force and internal stress distribution in augmented vertebrae.

The results demonstrated that increasing the stiffness of the augmentation material improved vertebral fracture strength, with the most substantial gains occurring up to an elastic modulus of 1000 MPa. Beyond this threshold, additional stiffness produced diminishing strength benefits and a disproportionately high increase in internal stress. Specifically, increasing stiffness from 300  to 2500 MPa led to only a 29.6% increase in fracture force but caused a 106% increase in internal stress. Importantly, materials around 300 MPa restored vertebral strength while maintaining stress distributions statistically similar to the intact bone (*p* > 0.05). Augmentation size also played a role, as larger augmentations (50%) benefitted more from material stiffness, whereas smaller augmentations (20%) showed less improvement in strength with stiffer materials but still experienced elevated stress levels.

Overall, a material with an elastic modulus in the range of 300–1000 MPa is identified as clinically safe and effective. The lower end of this range is more appropriate for weaker vertebrae or smaller defects, while the upper end is better suited for stronger bone or larger defects. Importantly, augmentation materials with lower stiffness (e.g., ~ 300 MPa) can restore vertebral strength while maintaining stress distributions closer to the intact condition. This balance is critical in clinical practice, as overly stiff cements such as PMMA may generate stress concentrations and increase the risk of adjacent-level fractures. By contrast, materials with stiffness values closer to cancellous bone may reduce such risks while still achieving adequate reinforcement. These findings support the use of patient-specific indicators, such as BMD or HU values, to tailor augmentation strategies and optimize outcomes. The developed QCT/FEA framework provides a low-cost, adaptable tool for preclinical evaluation, with the potential to be translated into a clinical setting. Moreover, the results can guide polymer developers in designing bone cements with stiffness properties optimized for patient-specific needs, ultimately supporting safer and more effective treatment strategies for patients with metastatic vertebral lesions.

## Data Availability

The datasets generated and/or analyzed during the current study are available from the corresponding author on request.
